# *JAC* Supplement: Survey of Antibiotic Resistance (SOAR) results 4—respiratory pathogen susceptibility

**DOI:** 10.1093/jac/dkaf292

**Published:** 2025-11-24

**Authors:** Alan Johnson, Jean-Yves Madec, Spyros Pournaras

**Affiliations:** Journal of Antimicrobial Chemotherapy, Birmingham, UK; Unité Antibiorésistance et Virulence Bactériennes, ANSES–Université de Lyon, Lyon, France; Clinical Microbiology Laboratory, Attikon University Hospital, Medical School, National and Kapodistrian University of Athens, Athens, Greece

## Foreword

Community-acquired respiratory tract infections (CA-RTIs) continue to pose a major global health challenge. Development of local or national treatment guidelines should ideally take account of rates of antimicrobial resistance in bacterial pathogens associated with CA-RTIs. However, such data are often not readily accessible. To help address this issue, the Survey of Antibiotic Resistance (SOAR), an international antimicrobial resistance surveillance programme, was established in 2002. SOAR collects data on the susceptibility of two key respiratory pathogens, namely, *Streptococcus pneumoniae* and *Haemophilus influenzae*, with a focus on countries where susceptibility data from other sources are not always widely available.

This Supplement provides an update on international surveillance data for resistance in *S. pneumoniae* and *H. influenzae* previously published in the Supplements of the *Journal of Antimicrobial Chemotherapy* in 2016, 2018 and 2020. In a series of eight articles, data are made available for countries in Europe (Italy, Spain, Ukraine), North Africa (Tunisia) and Asia (India, Kuwait, Pakistan, Turkey, the United Arab Emirates, Vietnam). Important resistance issues were observed among *S. pneumoniae* in several countries, commonly affecting oral/low-dose penicillin, while *H. influenzae* exhibited overall higher susceptibilities for most antibiotics except trimethoprim/sulfamethoxazole. Studies such as these are important in providing greater insight into the epidemiology of antimicrobial resistance over time at both a national and international level and guiding empiric therapy of infections.

We thank the authors for their contributions to this Supplement. Given the global scale of the threat posed by antibiotic resistance, it is hoped that the articles herein will be of interest to healthcare professionals, researchers, policymakers and others engaged in efforts to ensure bacterial infections remain treatable.

